# *The Organon* and Materia Medica Club of the Bay Cities of California

**Published:** 1896-06

**Authors:** W. E. Ledyard

**Affiliations:** Secretary


W. E. Ledyard, *Secretary.*

Reported by Eleanor F. Martin, M. D.

---

### *THE ORGANON* AND MATERIA MEDICA CLUB OF THE BAY CITIES OF CALIFORNIA.

The regular semi-monthly meeting was held at the office of Dr. J. M. Selfridge, in Oakland, Friday evening, January 17th, 1896.

Members present: Drs. J. M. Selfridge, W. E. Ledyard, M. F. Underwood, G. J. Augur, C. M. Selfridge, and George H. Martin.

The meeting was called to order at 8.15 o'clock by the President, Dr. J. M. Selfridge. The minutes of the previous meeting were read by the Secretary, and, after correction by Dr. J. M. Selfridge, were approved.

The President then appointed Dr. Martin reader of the evening, who read from *The Organon*, Sections 41–43.

DISCUSSION.

Dr. J. M. Selfridge—It does not seem possible that two diseases could be present in the body at the same time.

Dr. Martin—It would hardly seem possible.

Sections 44–46 were read.

DISCUSSION.

Dr. J. M. Selfridge—What a wonderful investigator Hahnemann was. How much he must have read in order to be able to explain all that he has!

Dr. Martin—We do not give Hahnemann one-half the credit he deserves. He certainly was a very learned man.

Sections 47–50 were read.

DISCUSSION.

Dr. Martin—This last section is one which contains food for a great deal of thought just at the present time. It distinctly says that if it were possible to inoculate a person with measles, it would be a dangerous thing to do if the patient were suffering with another skin disease, because the measles might affect the system, and the after results might be more severe than the primary disease. The same holds good in the Anti-toxine treatment. It is an element of a drug which annihilates a disease. In order to cure, the Anti-toxine must set up some morbific disease which may cure. If it does so, is it really a cure? It may cause more trouble than the disease we are trying to cure.

Dr. Augur—The dose given is too large, and the disease which is set up cannot be controlled.

Dr. Ledyard—As Dr. Augur says, the dose is too large.

Dr. C. M. Selfridge—While I was in Boston a patient in the hospital was suffering from diphtheria, and Dr. Kimball said it was the mildest of four cases. He used Anti-toxine, and that case was the only one that died.

Dr. Ledyard—I heard of a case in Santa Rosa; a little girl with diphtheria, who was very sick. She was cured by Antitoxine. The father told me that it seemed to be a wonderful cure.

Sections 51–54 were read.

### Discussion.

Dr. J. M. Selfridge—I want to remark in connection with this. We have to-day persons who call themselves homœopaths teaching that Homœopathy has limitations. Some time ago Dr. ——, a teacher of materia medica in a homœopathic college, read a paper in one of the societies in which he stated that there are limitations of the law of similars, and the editor of one of our journals has seconded this. With this state of affairs it is no wonder that Homœopathy is not progressing.

Dr. Martin—No one was more sorry than I to see that editorial. I made some remarks when the paper was read. I said that Dr. —— made a mistake in the title of the paper, which was: "Homœopathy a Specialty in Therapeutics." I said that he might call it a specialty if he would say that hygienic and surgical measures were also specialties. We know that Homœopathy or any other "pathy" is not going to cure an incurable case, or one in which there is great destruction of tissue. Hahnemann says this himself.

Dr. J. M. Selfridge—It is perfect heresy to say that Morphine must be used often.

Dr. Ledyard—The crude drug suppresses the symptoms, which are, or should be, the sole guide in the selection of the remedy.

Dr. Martin—The homœopathic remedy would act even if the Morphine were given.

Dr. Ledyard—The Morphine only relieves palliatively, suppressing the symptoms, and thus making it impossible to select the remedy, in consequence of the absence of symptoms.

Dr. J. M. Selfridge—Sometimes it is hard to get out of a patient symptoms that you can prescribe for, because there is so much pain that the patient cannot tell much about it. In one case I came near giving *Plumbum*, waited a little while and found that *Colocynth* was the remedy, which I gave, and it cured.

Sections 55–59 were read.

The President then declared the meeting adjourned to meet again the first Friday in February, at the office of Dr. George H. Martin in San Francisco.

